# Supplementary material for: Meta-Analysis of Survival Effects of Receptor Tyrosine Kinase-like Orphan Receptor 1 (ROR1)
Source: Medicina (Kaunas). 2022 Dec 17;58(12):1867. doi: 10.3390/medicina58121867 (PMC9784027; doi:10.3390/medicina58121867)
Supplement: Supplementary file 1 [file medicina-58-01867-s001.zip › medicina-2083481-supplementary.pdf]

Table S1: Summary of four studies in analysis for odds ratio related with cancer stage and lymph node metastasis.

First author (year) [ref]	Early stage (I/II)		Advanced stage (III/IV)		Lymph node metastasis (-)		Lymph node metastasis (+)	
	High ROR1	Low ROR1	High ROR1	Low ROR1	High ROR1	Low ROR1	High ROR1	Low ROR1
Zheng (2016) [33]	29	107	44	32	21	96	59	51
Zhou (2017) [35]	34	72	70	10	35	72	69	10
Mao (2019) [37]	37	63	25	16	3	15	32	19
Liu (2020) [39]	18	275	7	51				
